# Use of CIED Generated Heart Failure Risk Score (HFRS) Alerts in an Integrated, Multi-Disciplinary Approach to HF Management—A Prospective Cohort Study

**DOI:** 10.3390/s22051825

**Published:** 2022-02-25

**Authors:** Daniel Garner, Lindsay Lunt, Wing Leung, Jennifer Llewellyn, Matthew Kahn, David Jay Wright, Archana Rao

**Affiliations:** Liverpool Heart and Chest Hospital, Thomas Drive, Liverpool L14 3PE, UK; danielrobert.garner@lhch.nhs.uk (D.G.); lindsay.lunt@lhch.nhs.uk (L.L.); w.leung@nhs.net (W.L.); jennifer.llewellyn@lhch.nhs.uk (J.L.); matthew.kahn@lhch.nhs.uk (M.K.); david.wright@lhch.nhs.uk (D.J.W.)

**Keywords:** heart failure risk, device diagnostics, integrated care, outcomes

## Abstract

Aim: To evaluate use of CIED-generated Heart Failure Risk Score (HFRS) alerts in an integrated, multi-disciplinary approach to HF management. Methods: We undertook a prospective, single centre outcome study of patients implanted with an HFRS-enabled Medtronic CIED, generating a “high risk” alert between November 2018 and November 2020. All patients generating a “high risk” HFRS alert were managed within an integrated HF pathway. Alerts were shared with local HF teams, prompting patient contact and appropriate intervention. Outcome data on health care utilisation (HCU) and mortality were collected. A validated questionnaire was completed by the HF teams to obtain feedback. Results: 367 “High risk” alerts were noted in 188 patients. The mean patient age was 70 and 49% had a Charlson Comorbidity Score of >6. Mean number of alerts per patients was 1.95 and 44 (23%) of patients had >3 “high risk” alerts in the follow up period. Overall, 75 (39%) patients were hospitalised in the 4–6-week period of the alert; 53 (28%) were unplanned of which 24 (13%) were for decompensated HF. A total of 33 (18%) patients died in the study period. Having three or more alerts significantly increased the risk of hospitalisation for heart failure (HR 2.5, CI 1.1–5.6 *p* = 0.03). The feedback on the pathway was positive. Conclusions: Patients with “high risk” alerts are co-morbid and have significant HCU. An integrated approach can facilitate timely risk stratification and intervention. Intervention in these patients is not limited to HF alone and provides the opportunity for holistic management of this complex cohort.

## 1. Background (Introduction)

Heart failure (HF) is a complex clinical syndrome associated with significant morbidity and mortality [1]. Periods of decompensation drive health care utilization (HCU) [2] and diminish quality of life (QoL) [3]. In the UK, it is estimated that HF accounts for a total of 1 million inpatient bed days, 5% of all emergency medical admissions to hospital, and costs around GBP 2bn annually (2% of the total NHS budget). Two-thirds of these are attributed to HF with reduced ejection fraction (HFrEF) and these patients have significant comorbidity [4].

The ongoing coronavirus pandemic has emphasised the importance of ‘remote’ working, with the vast majority of HF patients in the vulnerable category and shielding. This has made patients reluctant to attend outpatient services in person [5].

Selected HFrEF patients benefit from Cardiac implantable electronic devices (CIEDs) (Cardiac Resynchronisation and/or Implantable Cardioverter Defibrillators) which have been proven to improve prognosis [1]. These devices provide ongoing monitoring (diagnostics) of the patients in addition to life saving pacing and defibrillation therapy.

In recent years, there has been growing interest in identifying strategies for early detection of disease progression to mitigate an individual patient’s risk of unplanned hospitalisation. Remote monitoring (RM) of HF patients utilising data from CIEDs is one approach that has been evaluated in this context. The results from the clinical trials and meta-analyses have failed to show improved clinical outcomes [6,7,8]. RM through therapeutic CIEDs has however shown promise in identifying heart failure patients at high risk of hospitalisation [9,10].

Questions about how technology can be used to ensure better, timely decision-making, rather than generate a higher workload need to be addressed [11]. The European Society of Cardiology (ESC) guidelines for HF recommend a multi-disciplinary approach for targeting intervention in high risk patients [12]. In selected patients, integration of HF and device services with a defined pathway and collaborative working could potentially streamline HF care and have a positive impact on patient outcomes [13].

In this paper, we review the impact of a pathway integrating device diagnostics with heart failure care, on patient outcomes.

## 2. Objectives

This single arm, prospective cohort study was aimed at assessing the impact of using ‘high risk’ HFRS alerts to guide HF care. We developed a collaborative, multidisciplinary approach to manage HF patients with Medtronic CIEDs, by sharing “high risk” HFRS alerts directly with the community HF teams.

## 3. Methods

### 3.1. Setting

We are a cardiac tertiary centre in the Northwest of England, responsible for the implant and follow up of complex CIEDs for a population of ~2 million. The follow up for these devices is performed via RM by Cardiac physiologists based at the tertiary centre supported by the Cardiologists. The majority of patients were monitored via a 6 monthly automated or manual remote download regimen with an annual face-to-face clinic review as per the Heart Rhythm Society/European Heart Rhythm Association recommendations [14]. RM is performed via CARELINK, a remote secure web-based platform for the Medtronic CIEDS.

HF care in the region is provided by multi-disciplinary teams comprising Heart Failure nurses (community and hospital), Consultant Cardiologists, and primary care physicians. The Community HF teams are geographically based and roughly aligned with the local Clinical Commissioning Groups (CCGs). A tertiary centre-based specialist HF and Device nurse has an overview of HF patients with CIEDs in situ and functions as a link nurse with the 8 community HF teams, comprising 40 nurses in total.

### 3.2. CIED Diagnostics

HFRS is a validated dynamic HF risk prediction tool available on selected Medtronic CIEDs, which uses physiological data to generate a risk score. Data stored within the HFRS-enabled device include: patient activity, AF/AT burden, heart rate variability, treated ventricular arrhythmia, percentage biventricular pacing, impedance/optivol, and night heart rate. HF Teams can be alerted via email and view online the actionable data. In the initial validation study (a post hoc analysis), the rate of HF hospitalization was 6.8% in the ‘high-risk’ HFRS group compared with only 0.6% in the low-risk HFRS group, a 10-fold increased risk [7,10].

### 3.3. Study Design

A single-arm prospective cohort study was undertaken according to the Standards for Reporting Diagnostic accuracy studies (STARD) guidelines [15]. The study duration was 2 years (November 2018–2020) and minimum follow up was 2 months (post alert).

### 3.4. Protocol

**Figure 1:**
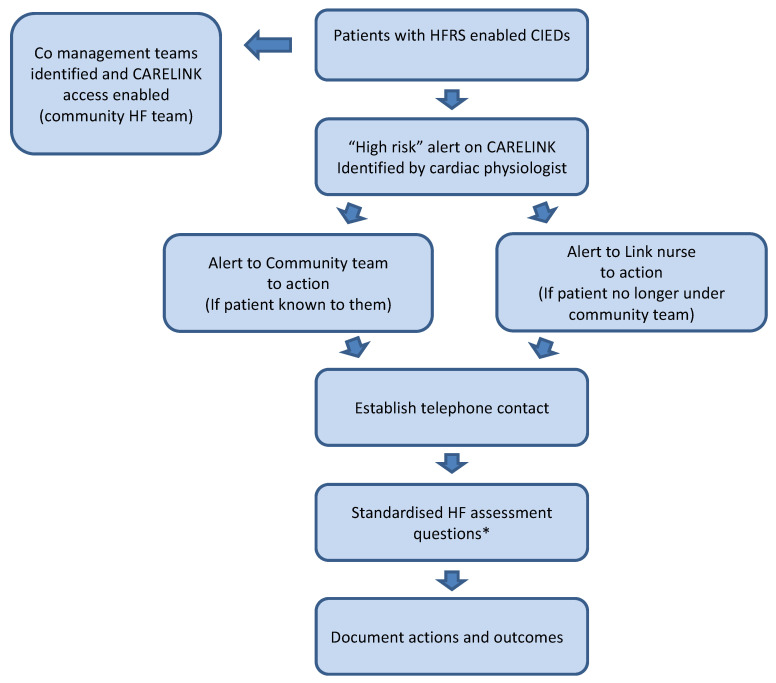
Study protocol. HFRS: Heart failure risk score, CIED: Cardiac Implantable Electronic Device, HF: Heart Failure. * See Appendix A for standardised HF questionnaire.

All patients on CARELINK with HFRS-enabled Medtronic CIEDs with a “high risk” alert were prospectively recruited over a 24-month period. When an alert was triggered, it was forwarded onto the relevant HF team (via co management clinic) for the individual patient (see Figure 1). Educational sessions were undertaken on an ongoing basis to familiarise the community HF teams with the device diagnostics, the HFRS and the clinical actions resulting from evaluation of these data.

### 3.5. Setting up of Co-Management Clinic

Virtual co-management clinics were set up on CARELINK for each CCG-based HF nurse group. Patients were allocated to these virtual clinics based on postcode. Access to CARELINK was organised for the community nurses and an ongoing support network established for the new users.

The HF/Devices nurse at the centre functioned as the link nurse between the centre and the community teams. Where patients were known to and under a Community team, their “High risk” alerts were shared directly with the team, and the teams were responsible for actioning these. The rest of the alerts were noted and actioned by the HF team at the centre. On receipt of the alert HF teams were to establish telephone contact and undertake standard HF assessment questions (see Appendix A) to assess clinical situation. In keeping with current practice, management was initiated and included alteration of medication, in person reviews, onward referral, or hospitalisation.

### 3.6. Outcomes

Primary outcomes included unplanned hospitalisation and death; secondary outcomes included healthcare utilisation (HCU) within 4–6 weeks of the high-risk alert. HCU was defined as any medical intervention in response to the alert including alteration of medication, device or onward referral and hospitalisation.

Two clinical frailty scores were also used, the Rockwood clinical frailty score (CFS) [16] and the Charlson Comorbidity Index (CCI) [17]. The CFS is a 9-point scale which provides a simple clinical measure of biological age and has been shown to correlate well with clinical outcomes [18]. The CCI is the most extensively studied comorbidity index and allocates a weighted score between 1 and 6 for each comorbidity. It estimates a 10-year survival rate and has also been shown to correlate with outcomes in heart failure patients [19].

A validated questionnaire was used to evaluate the team’s experience with the pathway [20]. We used the Health Optimum Telemedicine Questionnaire (see Appendix B). It has been found to be a robust predicative model of healthcare professional’s satisfaction with telemedicine programs. It includes 8 general questions for healthcare professionals to focus on their perception of the quality, convenience and technical challenges of the telemedicine service. Workload was assessed from feedback in these forms and discussions in steering meetings.

### 3.7. Data Collection

Patient details, medication, actions taken and outcomes including onward referral, hospitalisation and death were logged prospectively. To ensure completeness of data, all patient charts were reviewed retrospectively at the end of study. ForeCare (Share2Care) eXchange application was accessed to scan clinical documentation from the NHS trusts in the region to evaluate admissions within a 4–6-week period of the high alert and cross check data.

Dates of death were obtained via the NHS Demographic Batch Service (DBS), which links patient data to the NHS Spine (https://digital.nhs.uk/services/spine, accessed on 1 December 2020). The trace was performed, and all 188 unique patients included in the study returned a successful match to their Spine record. For those patients with follow up outside the region, the local HF clinicians were contacted for follow up information to ensure complete outcome data. Evaluation of the end user experience of the HFRS alerts and the technology interface was collected from the HF teams involved in the project. Regular steering meetings were undertaken to troubleshoot any issues and smooth collaborative working.

### 3.8. Statistical Analysis

Data were collected and inputted on Microsoft Excel and statistical analysis carried out on SPSS 26.0 (IBM Corporation, Armonk, NY, USA). Descriptive statistics were calculated and where normally distributed continuous variables are expressed as means with standard deviations. Categorical data were expressed as percentages. Cross tabulations, chi-squares and cox-regression analysis were used. The null hypothesis was rejected if *p* values were <0.05, and 95% confidence intervals were reported.

### 3.9. Ethics

The study was carried out in accordance with the Declaration of Helsinki. The Institutional Research Board at our centre reviewed the protocol and approved it. Patient consent for data collection and sharing was obtained at the time of enrolment for remote monitoring (CARELINK) of the CIEDs and was limited to usual care providers.

The team involved in the pathway were responsible for care of the patient and sought to leverage information from the CIED to help guide clinical management. On the basis that we routinely respond to CARELINK alerts in our practice, the integrated HF pathway would represent an extension of standard clinical practice. Specific consent for patient data to be used in this study was therefore not required.

## 4. Results

During the study period, we identified 749 patients with HFRS-enabled CIEDs on CARELINK. The device alerts were activated in clinic, and 564 of the 749 (75%) patients had automatic alerts turned on. The remainder were either not reached in time for the study commencement or opted not to have their alerts on. Even without the alert enabled, scheduled downloads and manual transmissions would still transmit a high HFRS at the time of scheduled automatic/patient initiated manual download and so these were included in the analysis. 367 high risk HFRS transmissions were received in 188 (25%) patients over a 24-month period between November 2018 and November 2020. (See Table 1) Median follow up was 14 months (range 2–26 months). The most common abnormal HFRS parameters were optivol (25%), reduced activity levels (24%), elevated night ventricular rate (18%) and low amount of biventricular pacing (13%).

The mean number of transmissions in patients who triggered alerts was 1.95 and 44 (23%) patients had >3 high alerts. The cohort was predominantly male (78%) with a mean age of 70.3 years. 176 (94%) had CRTDs in situ and 105 (56%) had an ischemic aetiology. 85% were on ACEi/ARB or ARNI, 93% on beta-blockers (BB) and 62% taking mineralocorticoid receptor antagonists (MRA). The population was expectedly co-morbid, 92 (49%) had CCI Score > 6 with a median score of 5 and the median CFS was 4 (See Table 1).

### 4.1. Patient Contact

Only 45 (24%) patients were under active follow up of community HF nurse teams; the remaining were contacted by the link nurse at our centre.

### 4.2. Response to “High Risk” Alerts

Contact was established in 303/367 (83%) of alerts. A total of 68 (23%) reported no symptoms. No intervention was required in 128 (35%) alerts (68 asymptomatic, 49 were improving clinically and 11 had previously been actioned). A total of 110 (31%) of the alerts resulted in a review by primary care team, HF nurse team or by palliative care team. A further 47 (13%) alerts generated a Cardiology clinic review. A total of 18 alerts were in inpatients (See Table 2).

### 4.3. Patient Outcomes

There were 53 unplanned hospital admissions (28%) within 6 weeks of the HFRS alert, with roughly half of these for HF decompensation (24 admissions). A total of 15 (8%) of patients had therapy from their device and received appropriate advice about driving and medication.

A total of 18 (10%) patients had advanced care planning initiated with ICD deactivation; 33 (18%) patients died during follow up.

**Figure 2:**
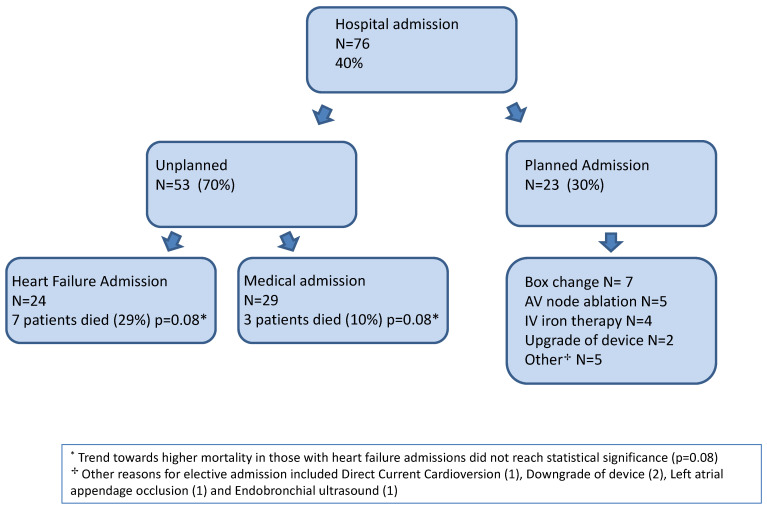
Flow chart of patient outcomes during study period.

The mortality of patients with unplanned admissions was 21%; there was a trend of higher mortality in those with HF admissions compared to non-HF admission, but this did not reach statistical significance (29% vs. 14% *p* = 0.16). A total of 23 (12%) patients had an elective admission and underwent AV node ablation (5 patients), routine generator replacement (7 patients), intravenous iron therapy (4 patients) and 2 patients had device/lead upgrades for increased functionality. (Other reasons for elective admission included 1 DCCV, 2 downgrades, 1 EBUS and 1 LAA occluder) (see Figure 2).

### 4.4. Factors Predicting Adverse Outcome 

**Figure 3:**
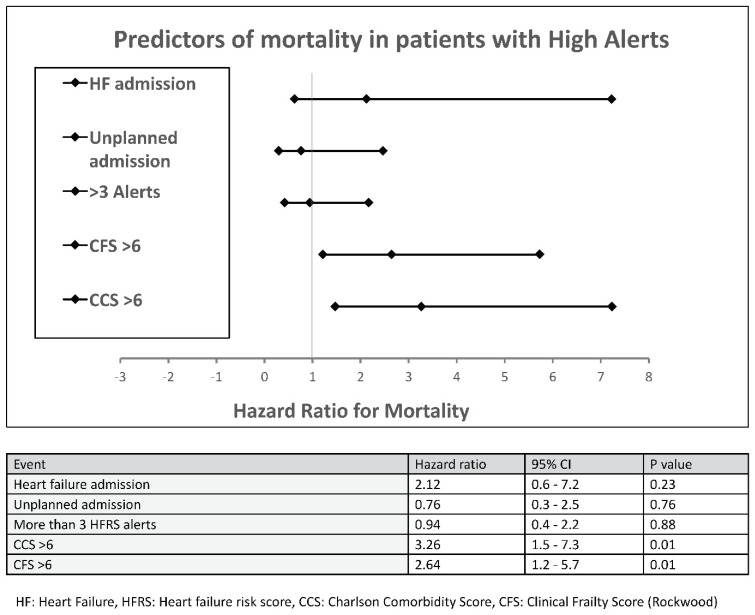
Predictors of mortality in patients with high-risk alerts. HF: Heart Failure, HFRS: Heart failure risk score, CCS: Charlson Comorbidity Score, CFS: Clinical Frailty Score (Rockwood).

Patients with >3 high risk alerts had a significantly higher likelihood of heart failure hospitalisation (HR 2.5, CI 1.1–5.6 *p* = 0.03). An elevated CCS and CFS score of >6 significantly increased the risk of death (HR 3.3, CI 1.5–7.2 *p* = 0.01 and HR 2.6, CI 1.2–5.7 *p* = 0.01, respectively). Heart failure admission resulted in a hazard ratio for death of 2.1 but confidence intervals were wide, and the *p* value was 0.23 (See Figure 3).

### 4.5. End User Experience

The end user feedback was obtained from all community teams involved in the project. The majority of users (75%) rated the technical quality of the user interface as “good”, and the quality of care delivered by the pathway as “comparable” or “better” than traditional service delivery models. Overall, 88% of the respondents thought that the pathway improved the health status of patients and importantly all respondents wished to continue using the service (either in the same way as it was initially deployed or with some improvements). Despite a seemingly large number of high-risk alerts, overall it translated to <4 alerts per week when distributed between the eight community teams and workload was not reported to be a significant issue.

## 5. Discussion

This paper describes the real-world outcomes of an innovative integrated pathway using CIEDs generated alerts to identify and intervene on selected HF patients. The “high risk” alerts were triaged and actioned by the HF team and the clinical impact of this evaluated. Whilst 128 (35%) of the alerts required no intervention, only 68 (18%) were truly asymptomatic; the rest were either recovering, had been intervened upon or required further review supporting the previously reported high sensitivity of “high risk” alerts in detecting not just decompensated HF but other conditions worthy of intervention [10].

A total of 76 (40%) of patients were hospitalised within 6 weeks of the alert; 53 (28%) were unplanned and 23 (12%) were planned for intervention to reduce further morbidity. This is the largest study of patients with high HFRS alerts, with the longest follow up, and the HF admission rate of 13% is similar to those in previous published studies [10,21]. The rate of unplanned hospitalisation of 28% is also less than the admission rate in the REM HF trial [8]. The outcome data suggest that these patients are vulnerable and have a high HCU across both primary and secondary care. There is little doubt that they would benefit from seamless care.

Our cohort was predominantly male (78%) with a mean age of 70 y, younger than the mean age of 78 y for HF in the UK [4]. A very high proportion of the study cohort (94%) had CRTD in situ, with a small percentage of ICD and CRTP. This is not reflective of our implanting practice (with older, frailer patients more likely to require CRTP) and the bias is predominantly to do with hardware availability and stock issues. As a predominantly two-company implanting centre, the majority of our low voltage devices were of a different manufacturer preceding and during the study period. This selection bias also explains the relatively younger age of our cohort. Background medical therapy was comparable to other contemporary studies with 85% of patients on ACEi/ARB/ARNI modifiers (the remainder intolerant of them), 93% on BB and 63% on MRA, reflecting optimised HF management [22].

A quarter (188/749) of the total HFRS-enabled CIED cohort generated “high risk” alerts in our population suggesting a sicker cohort. Despite the younger age, patients were relatively comorbid and just under 50% had a Charlson Comorbidity Score (CCS) of >6, which was predictive of increased mortality. This is in keeping with a study reporting a combination of device parameters (i.e., Fluid index, AF, and activity) can provide powerful incremental prognostic information in patients who are already at a particularly high risk of death based on the MAGGIC score [23].

A recent study that evaluated the HFRS algorithm to assess its accuracy in identifying worsening heart failure in a real-world cohort endorsed its use in the management of the ambulatory heart failure population [24]. The study proposed limiting evaluation to real-time automated HFRS alerts and acknowledged the value of outcome data in establishing the value of the clinical pathway. Our study confirms the value of HFRS in this population and provides insightful data into clinical outcomes.

Reports of increased mortality from COVID-19 in patients with underlying cardiovascular disease have resulted in HF patients shielding, with reduced attendance in cardiology outpatient departments [5]. HFRS alerts provide a unique opportunity to identify and manage those patients at highest risk and direct resources to those with greatest clinical need.

We found that the interventions resulting from the alerts ranged from alteration of medication to interventional procedures to improve therapy (upgrades, AV node ablations) to end of life discussions and deactivation of ICDs, spanning all aspects of HF management.

We propose that this reduced morbidity and improved outcomes in patients. Traditionally, HF and Device management have been followed up in parallel clinical streams by the HF team and the Cardiac Physiologists, respectively. Integrated HF care for CIED patients is the key to effective resource utilisation and this project is a step in this direction with integration of not just CIED and Heart Failure services but also within hospital and community settings.

Our pathway attempts to do this for a selected high risk HF cohort, with the use of existing resources but was somewhat thwarted by the fact that only 25% of patients were under regular community heart failure nurse follow up, having previously been discharged as “stable”. We believe that there is value in re-establishing links with community HF teams at the time of the CIED implant by setting up co-management clinics at the outset. The end user feedback from the HF team was overall positive, and a common theme was that the patients felt extremely reassured that they were being ‘monitored’ from afar allowing ease of access when necessary. This appears to be a user-friendly pathway which has become vital during the current pandemic.

### 5.1. Workload

The 367 alerts over a 24-month period translated to <4 alerts per week distributed between 8 teams, thus not conferring a significant workload burden. A total of 184 “high risk” alerts per year divided between 1 link nurse and 40 community nurses, i.e., ~<5 “high risk” alerts per year per nurse. This meant some users felt that they became ‘unfamiliar’ with the pathway despite provision of ongoing technical and clinical support. This was to do with the infrequent number of high alerts that a member of a team may be called to act on; whilst this may be a good thing for clinical workload, it did result in a general reluctance to engage with the CARLINK data.

The MORE CARE study [25] suggested that in their experience 10% of all alerts were “high risk” alerts, thus a possible solution may be to have the team look at but not action both “low risk” and “medium risk” alerts as way of keeping themselves upskilled. Alternatively, a regular remote meeting to discuss High risk cases on a monthly basis may be useful to keep the key stakeholders engaged and informed.

### 5.2. Limitations

The lack of a control arm makes it difficult to gauge the true impact of the pathway on patient outcomes. The patients in our cohort were limited to those with HFRS-enabled Medtronic CIEDs and, whilst we cannot extrapolate our findings to a similar cohort with other manufacturer CIEDs, there is no reason to believe an integrated pathway facilitating device guided HF management would not benefit them.

We acknowledge that “asymptomatic” patients with a high-risk alert may go on to develop symptoms at a later stage, but a closer monitoring regime would require a greater investment of time and resources and needs to be evaluated in the further studies. The small number of CRTPs (5%) in this cohort represent a missed opportunity as we believe that this population is likely to be older, frailer and even more likely to benefit from integrated care.

### 5.3. Conclusions

An integrated approach to HF for patients with CIEDs in situ can facilitate risk stratification and intervention. We found the HFRS tool useful in identifying patients at risk of increased HCU. Intervention in these patients is not limited to HF care alone and provides the opportunity for holistic management of this complex cohort.

## Figures and Tables

**Table 1:** Demographics of the study population.

	No of Patients (*n* = 188)	
Male	147	78%
Female	41	22%
Mean age (±SD)	70.3 years	±11.5
Aetiology		
Ischaemic cardiomyopathy	105	56%
Non-Ischaemic	74	49%
Congenital/valvular	9	5%
Device		
CRTD	176	94%
CRTP	9	5%
ICD	3	1%
Number of alerts	365	
Mean alerts per patient	1.9	
Patients with single alert	101	54%
Patients with 2 alerts	43	23%
>3	44	
Medical therapy		
ACE/ARB	126	67%
ARNI	34	18%
Beta blocker	175	93%
MRA	116	62%
Diuretic	135	72%
Diabetes	71	38%
Mean BMI (±S.D)	29.6	±6.2
Clinical frailty score		
Mean score (±S.D)	4.1	±1.5
>6	25	14%
Charleson Comorbidity score		
Mean score (±S.D)	5.5	±2.3
>6	92	49%

**Table 2:** Outcomes.

Responses to High Risk Alerts	*n* = 367	
Telephone contact made	303	83%
No intervention required	128	35%
Asymptomatic	68	19%
Cardiac compass improving	49	13%
Alert previously actioned	11	3%
Reviewed by Heart failure nurses	85	23%
Referral to cardiology for review	47	13%
Referral to GP to further action	21	6%
Referral to palliative care	4	1%
Inpatient during alert	18	5%
Patient outcomes	*n* = 188	
Unplanned hospital admission	53	28%
Heart failure admission	24	13%
Death	33	18%
Elective admission	23	12%
AV node ablation	5	3%
Upgrade of device	2	1%
Box change	7	4%
IV iron therapy	4	2%
Other (DCCV, downgrade, LAAO, EBUS)	5	3%
Device therapy	15	8%
Device deactivation	18	10%

DCCV—direct current cardioversion; LAAO—Left atrial appendage occluder; EBUS—Endobronchial ultrasound.

## Data Availability

The gathered for this article will be shared on reasonable request to the corresponding author. No patient identifiable data will be made available, and all data will be anonymised.

## Appendix A

Standardised HF assessment questions:

1. Have you experienced any new or worsening breathlessness?
2. Have you experienced any new or worsening leg swelling?
3. Have you gained any weight?
4. Have you experienced any new or worsening fatigue?
5. Have you been experiencing any recent ill health or visited a doctor or nurse about anything else?

## Appendix B

Original EU Project Health Optimum Telemedicine Acceptance Questionnaire [20].

1. **How do you rate the overall quality of the telemedicine consultation?**
  1. Excellent
  2. Good
  3. Fair
  4. Poor
2. **How would you rate the technical quality of the telemedicine consultation?**
  1. Excellent
  2. Good
  3. Fair
  4. Poor
3. **How do you rate the quality of care delivered by the telemedicine service when compared to the quality of traditional care?**
  1. Better
  2. About the same
  3. Not as good
  4. Not sure
4. **Were you comfortable during the telemedicine consultation?**
  1. Yes, very comfortable
  2. Yes, somewhat comfortable
  3. No, somewhat uncomfortable
  4. No, very uncomfortable
5. **Do you feel that the telemedicine consultation service may influence the health status of your patients?**
  1. Improved health
  2. No change
  3. Negative effects on health
6. **Did you experience technical difficulties that might affect the quality of care delivered by the telemedicine service?**
  1. Not at all
  2. Sometimes
  3. Often
7. **Did you experience organisational or other difficulties that might affect the quality of care delivered by the telemedicine service?**
  1. Not at all
  2. Sometimes
  3. Often
8. **Would you continue to use the telemedicine service?**
  1. Yes, in the same way as the service has been deployed
  2. Yes, but with improvements
  3. No
